# Tunicamycins from Marine-Derived *Streptomyces bacillaris* Inhibit MurNAc-Pentapeptide Translocase in *Staphylococcus aureus*

**DOI:** 10.3390/md22070293

**Published:** 2024-06-26

**Authors:** Jayho Lee, Ji-Yeon Hwang, Daehyun Oh, Dong-Chan Oh, Hyeung-geun Park, Jongheon Shin, Ki-Bong Oh

**Affiliations:** 1 Department of Agricultural Biotechnology, College of Agriculture and Life Sciences and Natural Products Research Institute, Seoul National University, Seoul 08826, Republic of Korea; jayho@snu.ac.kr; 2 Natural Products Research Institute, College of Pharmacy, Seoul National University, Seoul 08826, Republic of Korea; yahyah7@snu.ac.kr (J.-Y.H.); dongchanoh@snu.ac.kr (D.-C.O.); 3 Research Institute of Pharmaceutical Sciences, College of Pharmacy, Seoul National University, Seoul 08826, Republic of Korea; oh1see@snu.ac.kr (D.O.); hgpk@snu.ac.kr (H.-g.P.)

**Keywords:** tunicamycins, *Streptomyces bacillaris*, antibacterial activity, *Staphylococcus aureus*, MurNAc-pentapeptide translocase

## Abstract

Four tunicamycin class compounds, tunicamycin VII (**1**), tunicamycin VIII (**2**), corynetoxin U17a (**3**), and tunicamycin IX (**4**), were isolated from the culture broth of the marine-derived actinomycete *Streptomyces* sp. MBTG32. The strain was identified using the 16S rDNA sequencing technique, and the isolated strain was closely related to *Streptomyces bacillaris*. The structures of the isolated compounds were elucidated based on spectroscopic data and comparisons with previously reported NMR data. Compounds **1**–**4** showed potent antibacterial activities against Gram-positive bacteria, especially *Staphylococcus aureus,* with MIC values of 0.13–0.25 µg/mL. Through a recombinant enzyme assay and overexpression analysis, we found that the isolated compounds exerted potent inhibitory effects on *S. aureus* MurNAc-pentapeptide translocase (MraY), with IC_50_ values of 0.08–0.21 µg/mL. The present results support that the underlying mechanism of action of tunicamycins isolated from marine-derived *Streptomyces* sp. is also associated with the inhibition of MraY enzyme activity in *S. aureus*.

## 1. Introduction

The prevalence of antibiotic-resistant bacteria has emerged as a major threat to human health, as infections from these bacteria are uncontrollable with commercial antibiotics [1]. Among the antibiotic-resistant bacteria, some are multidrug-resistant. Methicillin-resistant *Staphylococcus aureus* (MRSA) is a major pathogenic drug-resistant bacteria [2]. Drug resistance is caused by the selection of resistant organisms in antimicrobial conditions and the transfer of these resistance genes through mobile genetic elements, such as plasmids, bacteriophages, or transposons [3]. The acquisition of drug resistance is much faster than the rate of development of new antibacterial agents, but the investment in antibacterial drugs continues to decline [4]. As the number of newly discovered antibiotics is also decreasing, scientists have searched for new bioactive secondary metabolites from marine actinomycetes because their unique metabolisms might lead to structurally novel compounds [5,6]. As a result, various novel bioactive compounds have been discovered from rare marine actinomycetes [7].

Tunicamycins are a class of nucleoside sugar analog compounds that are characterized by the uracil moiety followed by an eleven-carbon dialdose sugar with an *N*-acetyl-D-glucosamine and an *N*-linked fatty acid residue [8]. Tunicamycins were first isolated from *Streptomyces lysosuperificus* as a mixture of homologs and later isolated as tunicamycin I, II, III, IV, and V [9,10]. Streptovirudins and corynetoxins, whose structures are similar to that of tunicamycins but differ in the *N*-linked acyl chains, were isolated from *Streptomyces griseoflavus* and *Corynebacterium rathayi*, respectively [11]. Tunicamycins are known to inhibit MurNAc-pentapeptide translocase (MraY), which is an essential membrane-associated enzyme in bacteria [12]. MraY transfers uridine diphosphate-MurNAc-pentapeptide to the lipid carrier bactoprenol phosphate (C55-P) to form lipid I, which is an intermediate of peptidoglycan synthesis [13,14]. MraY is a promising drug target for developing new antibacterial agents, though it is yet to be utilized as a target for commercial antibiotics [15]. Although tunicamycins have strong antibacterial activities against some Gram-positive bacteria—especially *S. aureus*—these compounds also inhibit dolichyl-phosphate *N*-acetylglucosamine-phosphotransferase 1 (DPAGT1) and show cytotoxicity in mammalian cells by damaging the membrane, leading to necroptosis [16,17]. While low specificity and cytotoxicity limited the use of tunicamycins as clinical drugs, recent attempts to achieve higher specificity toward DAPGT1 or antibacterial activity while lowering cytotoxicity has shown modified tunicamycins to be potential anticancer and antibacterial agents [17,18].

In the course of searching for bioactive secondary metabolites from marine-derived actinomycetes, we isolated the MBTG32 strain from marine sediment samples collected from Jeju Island, Republic of Korea. The strain was identified as *Streptomyces bacillaris* on the basis of its 16S rDNA sequence. This strain’s organic extract of a liquid culture exhibited potent antibacterial activity against *S. aureus* ATCC6538p with a minimum inhibitory concentration (MIC) of 0.5 μg/mL. Antibacterial-activity-guided separation of the extract using diverse chromatographic methods led to the isolation of four compounds: tunicamycin VII (**1**), tunicamycin VIII (**2**), corynetoxin U17a (**3**), and tunicamycin IX (**4**). We herein report the antibacterial activities of compounds **1**–**4** against several representative pathogenic bacteria, including *S. aureus* ATCC6538p and MRSA strains CCARM3090, ATCC43300, and ATCC700787. We also analyzed the mode of action of the compounds isolated through a MraY enzyme activity assay and overexpression assay.

## 2. Results and Discussion

### 2.1. Taxonomy and Phylogenetic Analysis of MBTG32

The phylogenetic analysis of the MBTG32 strain was conducted by amplifying the 16S rDNA sequence using polymerase chain reaction (PCR). The amplified sequence (1492 bp) was analyzed using the Basic Logic Alignment Search Tool (BLAST), and the sequence from the MBTG32 strain showed 100% identity with *Streptomyces bacillaris* strain NBRC13487 (GenBank accession number: NR041146). Therefore, this strain was designated as *Streptomyces bacillaris* strain MBTG32 (GenBank accession number: PP669811).

### 2.2. Isolation and Structural Elucidation of Compounds ***1***–***4***

**Figure 1:**
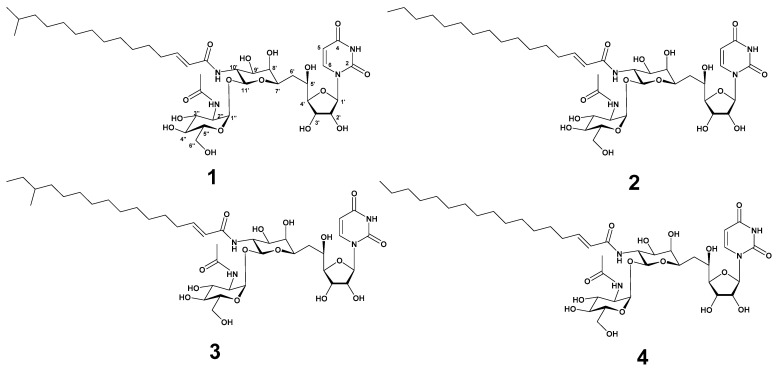
The structure of tunicamycin VII (**1**), tunicamycin VIII (**2**), corynetoxin U17a (**3**), and tunicamycin IX (**4**).

Strain MBTG32 was cultivated in GTYB broth and incubated for 7 days with shaking. The culture filtrate was extracted with *n*-hexane, and the aqueous fraction was subsequently extracted with ethyl acetate. The ethyl acetate fraction exhibited antibacterial activity against *S. aureus,* and further separation was conducted with C_18_ reversed-phase vacuum flash chromatography. After fractionation, the 100% methanol fraction was separated using semi-preparative high-pressure liquid chromatography (HPLC) to yield four compounds in the form of white powders. Based on spectroscopic analysis, including high-resolution electrospray ionization mass spectrometry (HR-ESI-MS), ^1^H, and ^13^C nuclear magnetic resonance (NMR), compounds **1**–**4** were identified as tunicamycin VII (**1**) [19,20], tunicamycin VIII (**2**) [20,21], corynetoxin U17a (**3**) [22], and tunicamycin IX (**4**) [20,21] (Figure 1). The acyl *iso* branching in compound **1** was determined from ^1^H NMR spectra, which showed a doublet (6H, *J* = 6.6 Hz, -CH(CH_3_)_2_) at δ_H_ 0.84. The presence of a linear acyl chain in compounds **2** and **4** was supported by ^1^H NMR spectrum at δ_H_ 0.80 (3H, t, *J* = 6.9 Hz) and δ_H_ 0.80 (3H, t, *J* = 7.0 Hz), respectively. In the ^1^H NMR spectrum of **3,** the presence of the acyl *anteiso* branching was confirmed from one doublet and one triplet methyl proton at δ_H_ 0.82 (3H, d, *J* = 6.2 Hz) and δ_H_ 0.83 (3H, t, *J* = 7.0 Hz). These assignments were further supported by HR-ESI-MS data and notable fragmentation patterns. HR-ESI-MS analysis of compounds revealed a mass distribution and fragmentation pattern identical to those of tunicamycins [20]. Molecular ions were detected as [M + H]^+^, along with sodium adducts [M + Na]^+^, and two diagnostically useful fragment ions [M + H − 221]^+^ and [M + H − 203]^+^ (Figures S14–S17). The spectroscopic data of these compounds were in good agreement with those in the previous literature (Figures S1–S17, Tables S2 and S3).

### 2.3. Antibacterial Activities of Compounds ***1***–***4***

Tunicamycin from *Streptomyces lysosuperficus* was previously reported to show antibacterial activity against several Gram-positive bacteria (MIC = 0.1–20 µg/mL), particularly those in the *Bacillus* genus [9]. Therefore, the antibacterial activities of compounds **1**–**4** were evaluated against six representative pathogenic bacterial strains. These compounds showed significant antibacterial activities against Gram-positive bacteria, especially *S. aureus* ATCC6538p, with MICs in the range of 0.06–0.25 μg/mL (Table 1). However, these compounds showed weak or no activities against Gram-negative bacteria. Based on the reported data, compounds **1**–**4** were further evaluated against various methicillin-sensitive *Staphylococcus aureus* (MSSA) and MRSA strains (Table 2). In the case of MSSA strains, compounds **1**–**4** exhibited potent antibacterial activities against CCARM0204 and CCARM0205 but substantially weak activities against CCARM0024 and CCARM3640. In the case of MRSA strains, compounds **1**–**4** did not show inhibitory activities against CCARM3089 and ATCC700788 and showed moderate to weak activities against CCARM3090, CCARM3634, CCARM3635, ATCC43300, and ATCC700787, with MIC values ranging from 2 to 64 μg/mL. These results indicate that the susceptibility of compounds **1**–**4** differs within *S. aureus* strains.

### 2.4. Effects of Compounds ***1***–***4*** on MraY Expression and Preparation of Recombinant MraY

**Figure 2:**
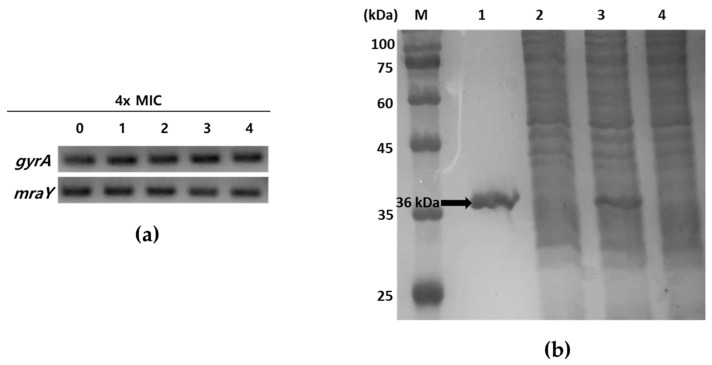
Analysis of *mraY* expression in the presence of compounds **1**–**4** and SDS-PAGE of recombinant MraY. (**a**) Semi-quantitative RT-PCR of *mraY* in *S. aureus* ATCC6538p. A housekeeping gene *gyrA* was used as a loading control. **0**: no compounds treated; **1**–**4**: treatment with four times MIC of compounds **1**–**4**, respectively. (**b**) SDS-PAGE analysis of purification of MraY. The samples were loaded on a 15% polyacrylamide gel. M: protein molecular weight standard; lane1: Ni^2+^-NTA bound proteins eluted with 250 mM imidazole; lane2: host with pET28a induced with 1 mM IPTG; lane3: host with pET28a-*mraY* induced with 1 mM IPTG; lane4: uninduced host with pET28a-*mraY*. The arrow indicates purified His6-tagged recombinant MraY (ca. 36 kDa).

Tunicamycins, liposidomycins, capuramycins, mureidomycins, and muraymycins are known as inhibitors of MraY, the first membrane protein in peptidoglycan formation, which is essential for bacterial growth [13,23,24]. To ascertain whether tunicamycins inhibit the transcription of *mraY* or block the function of MraY at the enzyme level without affecting the expression of the *mraY* gene, the mRNA expression levels of *mraY* in response to tunicamycins were initially explored using semi-quantitative reverse-transcription PCR (RT-PCR). *S. aureus* ATCC6538p was grown to mid-log phase in MHB media and treated with four times the MIC of compounds **1**–**4** at 37 °C for 40 min. Total RNA isolated from *S. aureus* ATCC6538p was reverse-transcribed into cDNA, and the *mraY* gene was amplified using this cDNA as a template. As shown in Figure 2a, *S. aureus* ATCC6538p showed no inhibition of *mraY* gene transcription in response to treatment with compounds **1**–**4**. Next, to determine the enzymatic activity inhibition of compounds **1**–**4**, recombinant MraY derived from *S. aureus* ATCC6538p was prepared and purified (Figure 2b). *S. aureus* 6538p *mraY* was cloned into pET28a to obtain recombinant plasmid pET28a-*mraY*. The recombinant His6-tagged MraY enzyme was overexpressed in *Escherichia coli* BL21 with 1 mM IPTG and purified from the detergent-solubilized crude extract by binding with Ni^2+^-NTA-agarose resin and eluting with 250 mM imidazole. The elution contained the MraY enzyme, which showed a single band in lane 1 at the calculated size of 36 kDa in SDS-PAGE (Figure 2b).

### 2.5. In Vitro MraY Enzyme Inhibition Assay

**Figure 3:**
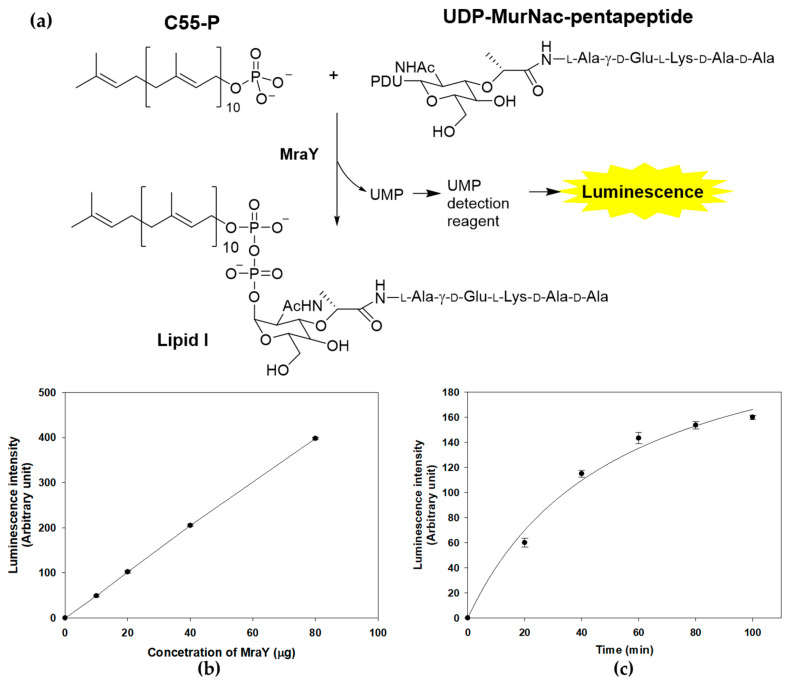
MraY enzyme reaction and activity measurement using UMP detection. (**a**) MraY catalyzes the reaction of C55-P and UDP-MurNAc-pentapeptide to form Lipid I. The enzyme activity is measured by the luminescence of the luciferase reaction from the UMP detection reagent. (**b**) Concentration-dependent activity of the MraY enzyme. The enzyme reaction was carried out under standard assay conditions at 37 °C for 1 h. (**c**) Time course of the MraY enzyme reaction. Reactions were carried at 37 °C in 100 μL, containing 100 mM Tris-HCl, 500 mM NaCl, 10 mM MgCl_2_, 20 mM CHAPS, 150 μM UDP-MurNAc-pentapeptide, 250 μM C55-P, and 30 μg of purified MraY.

The enzymatic activity of MraY can be accurately and quantitatively assessed through a sensitive discontinuous coupled assay system, where the luminescence output serves as a reliable indicator of enzymatic activity. MraY transfers UDP-MurNAc-pentapeptide to C55-P, resulting in the formation of lipid I and the release of UMP, the byproduct of MraY reactions [15]. The enzyme activity of purified recombinant MraY was measured by detecting the generated UMP with a UMP-Glo^TM^ glycosyltransferase assay (Figure 3a) [25]. In the presence of both substrates and purified MraY, an increase in luminescence was observed, indicating that a UMP-generating reaction was detected (Figure 3b). The activity was dependent on the amount of enzyme and the incubation time, suggesting that the assay system can be used to measure MraY activity in vitro (Figure 3b,c).

**Figure 4:**
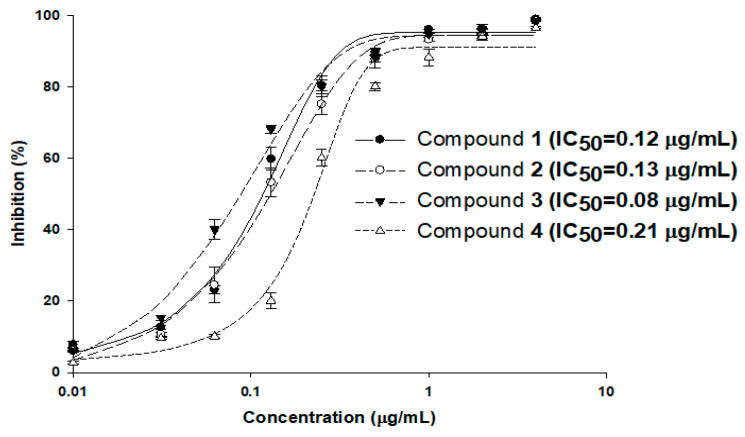
MraY enzyme inhibition by compounds **1**–**4**. The enzyme reactions were carried out three times under standard assay conditions, and the calculated IC_50_ values of compounds **1**–**4** were 0.12, 0.13, 0.08, and 0.21 μg/mL, respectively.

To evaluate the inhibitory activity of compounds **1**–**4** against MraY using the established luminescence assay system, MraY enzyme reactions were carried out with the addition of compounds in various concentrations in the range of 0.03 to 4 μg/mL, and the half-maximal inhibitory concentration (IC_50_) values were calculated (Figure 4). The reactions were carried out three times, and the calculated IC_50_ values of compounds **1**–**4** were 0.12, 0.13, 0.08, and 0.21 μg/mL, respectively. As the IC_50_ values of these compounds were in a similar range to that of the MIC values against *S. aureus* 6538p (0.13, 0.13, 0.06, and 0.25 μg/mL, respectively), MraY may be the primary target of the tunicamycins in *S. aureus*. The tendencies of the IC_50_ of MraY and antimicrobial activity were distinct among the MraY inhibitors. For instance, liposidomycins showed MraY inhibition with an IC_50_ at 0.03 μM and demonstrated antibacterial activity against Gram-positive bacteria. The MIC for *Mycobacterium phlei* was 1.6 μg/mL and did not show toxicity in mice [13,26]. Capuramycin strongly inhibited MraY (MurX) with an IC_50_ of 0.010 µg/mL and displayed selective antibacterial activity against mycobacteria. The MIC values were about 6.25 to 12.5 µg/mL for *M. smegmatis* [11]. Muraymycin A1 exhibited antibacterial activity against Gram-positive bacteria with MIC values of 2–16 µg/mL against *Staphylococcus* spp. It also showed therapeutic efficacy in *S. aureus*-infected mice at an effective dose of 1.1 mg/kg [24]. Further investigation is necessary to uncover the pharmacological properties observed among nucleoside inhibitors targeting MraY, as this aspect remains poorly understood.

### 2.6. MraY Overexpression In Vivo

**Figure 5:**
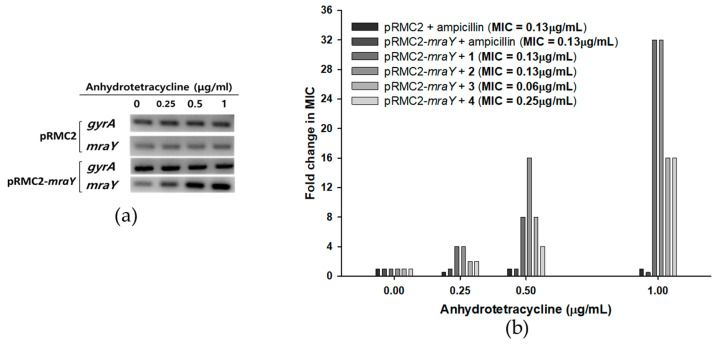
Effects of *mraY* overexpression in *S. aureus* 6538p cells on the MICs of compounds **1**–**4**. (**a**) Semi-quantitative RT-PCR of *mraY* in *S. aureus* bearing pRMC2 or pRMC2-*mraY* plasmids induced with anhydrotetracycline. Anhydrotetracycline was treated for 1 h, and overexpression of *mraY* was observed in *S. aureus* with pRMC2-*mraY,* while *mraY* in *S. aureus* with pRMC2 was not overexpressed. A housekeeping gene, *gyrA,* was used as a loading control. (**b**) *S. aureus* MIC fold change with *mraY* overexpression. Anhydrotetracycline was added in concentrations of 0.25, 0.5, and 1 μg/mL, and the MICs of compounds **1**–**4** were tested.

Since MraY is crucial for the survival of bacteria but absent in eukaryotes, it presents an excellent target for the development of novel antibacterial agents [13,17,27]. As MraY is an enzyme that is essential for the growth of *S. aureus*, deletion mutants could not survive without complementary copies of *mraY* [28]. In this case, overexpression studies have attempted to monitor the change in susceptibility to overexpressed conditions to validate the targets of inhibitors [29,30]. An overexpression study of *mraY* in *S. aureus* was recently conducted, showing that a synthetic compound, 8-octyl berberine, inhibits *S. aureus* peptidoglycan synthesis and that the overexpression of *mraY* resulted in a 16-fold increase in the MIC [31]. To further investigate the activity of tunicamycins with respect to MraY in vivo, an overexpression analysis of *mraY* was conducted by cloning the gene into a tetracycline-inducible plasmid pRMC2 to obtain a recombinant plasmid pRMC2-*mraY* [32]. The recombinant plasmid pRMC2-*mraY* was transformed into *S. aureus* 6538p, resulting in a tetracycline-inducible *mraY* overexpression system. Anhydrotetracycline, a less potent analog of tetracycline, was used for induction, and the overexpression of *mraY* was confirmed with semi-quantitative RT-PCR (Figure 5a). The MIC of anhydrotetracycline in *S. aureus* 6538p was 8 μg/mL, whereas the MIC of tetracycline was 0.13 μg/mL. *S. aureus* bearing the pRMC2-*mraY* plasmid was induced with anhydrotetracycline in the concentrations of 0.25, 0.5, and 1 μg/mL. The overexpressed *S. aureus* cells were collected to measure the MIC of compounds **1**–**4**. In *mraY*-overexpressed conditions, the MIC of compounds **1**–**4** showed concentration dependency toward anhydrotetracycline (Figure 5b). At 0.25 μg/mL anhydrotetracycline, the MIC of compounds **1** and **2** increased four-fold, and the MIC of compounds **3** and **4** increased two-fold. As the concentrations of anhydrotetracycline were increased to 0.5 and 1 μg/mL, the MIC of compounds **1**–**4** further increased 32-fold in the cases of compounds **1** and **2** and 16-fold in the cases of compounds **3** and **4**. The overexpression of *mraY* in *S. aureus* resulted in far less potency of compounds **1**–**4**, with up to a 32-fold increase in the MIC with respect to that in uninduced conditions. The MIC of ampicillin did not increase in any overexpressed conditions in either pRMC2 or pRMC2-*mraY,* meaning that the MIC change was specifically observed in compounds **1**–**4**. The MIC fold changes in MraY overexpression conditions show that *S. aureus* can overcome tunicamycin susceptibility when the MraY expression level is artificially increased; this result was similar to those of previously reported MraY overexpression tests of 8-octyl berberine [31], indicating that the antimicrobial activity of compounds **1**–**4** was related to inhibition of MraY in vivo. While extensive research is necessary to fully understand the effects of MraY overexpression in *S. aureus*, the overexpression system outlined in this study holds potential for evaluating the in vivo activity of MraY inhibitors.

## 3. Materials and Methods

### 3.1. General Experimental Procedures

^1^H and ^13^C NMR spectra were recorded using a Bruker Avance 600 MHz instrument (Billerica, MA, USA) in both MeOH-*d*_4_ and DMSO-*d_6_* solutions. High-resolution electrospray ionization mass spectrometry (HR-ESI-MS) data were acquired at the National Instrumentation Center for Environmental Management (Seoul, Republic of Korea) using an AB Sciex 5600 QTOF HR-MS instrument (Sciex, MA, USA). HPLC separations were conducted on a SpectraSYSTEM p2000 equipped with a UV-Vis detector (Gilson, Middleton, WI, USA). All solvents used were either of spectroscopic grade or were distilled prior to use. Substrates and antibiotics were from Sigma-Aldrich (St Louis, MO, USA) if not otherwise mentioned.

### 3.2. Taxonomic Identification of the Tunicamycin-Producing Microorganism

The bacterial strain MBTG32 was isolated from a marine sediment sample obtained from a seashore on Jeju Island, Republic of Korea. The strain identification was confirmed by sequencing the internal transcribed spacer region after PCR amplification of extracted DNA. Genomic DNA was extracted from MBTG32 mycelium using the i-Genomic BYF DNA Extraction Mini Kit (Intron Biotechnology, Seoul, Republic of Korea) following the manufacturer’s protocols. PCR amplification of the 16S rDNA sequence was accomplished under optimized conditions using primers 27F and 1429R (Table S1). The obtained nucleotide sequence was deposited in GenBank under the accession number PP669811.

### 3.3. Cultivation, Extraction, and Isolation

The MBTG32 strain was streaked to sporulate on GTYB agar plates composed of 10 g of glucose, 2 g of tryptone, 1 g of yeast extract, 1 g of beef extract, and 20 g of agar in 1 L of artificial seawater and incubated for 5 days at 28 °C. Mature spores were then transferred to 25 mL of GTYB broth and incubated for 24 h at 28 °C on a rotary shaker. Subsequently, each seed culture was scaled up to 500 mL of GTYB broth and incubated for 7 days at 28 °C on a rotary shaking incubator at 230 rpm. The resulting culture filtrate (40 L) was partitioned using equal volumes of *n*-hexane (0.6 g) and ethyl acetate (3.17 g).

The ethyl acetate fraction was separated by C_18_ reversed-phase vacuum flash chromatography using a sequential mixture of methanol and water as eluents (seven fractions in the gradient, water–methanol, from 100:0 to 0:100). The fraction eluted with 100% methanol which exhibited inhibitory activity against *S. aureus* (MIC = 0.26 μg/mL) was further separated with semi-preparative reversed-phase HPLC (Agilent C_18_ column, 10 × 250 mm; flow rate: 2.0 mL/min; water–acetonitrile, 50:50), yielding compounds **1** (*t*_R_ = 13.2 min), **2** (*t*_R_ = 14.1 min), **3** (*t*_R_ = 18.9 min), and **4** (*t*_R_ = 22.0 min). Additional purification of compounds **1** and **2** was conducted using analytical HPLC (Agilent C_18_ column, 4.6 × 250 mm; flow rate: 0.7 mL/min; water–methanol, 20:80), while compounds **3** and **4** were purified using analytical HPLC (Agilent C_18_ column, 4.6 × 250 mm; flow rate: 0.7 mL/min; gradient water–acetonitrile, 58:42 to 53:47 for 70 min). The isolated metabolites were obtained in quantities of 8.3, 4.5, 6.4, and 3.2 mg for compounds **1**–**4**, respectively.

### 3.4. Antibacterial Activity Assays

Antibacterial activity assays were conducted by following the guidelines outlined by the Clinical and Laboratory Standards Institute [33]. The bacterial strains used in these assays were from the Culture Collection of Antimicrobial Resistant Microorganisms (CCARM) at Seoul Women’s University (Seoul, Republic of Korea) and the American Type Culture Collection (ATCC) (see Table 1 and Table 2). The cells were cultured in Mueller–Hinton broth (MHB) at 37 °C for 16 h, collected via centrifugation, and washed with sterile distilled water. The test compounds were dissolved in DMSO and diluted two-fold with MHB from 0.016 to 128 μg/mL. DMSO was added to each well to give a final 1% DMSO concentration. Then, 90 μL of MHB containing the test compound was mixed with 10 μL of broth of the test bacterium containing 5 × 10^6^ colony-forming units (cfu)/mL in each well of a 96-well plate (final concentration, 5 × 10^5^ cfu/mL). The plates were then incubated for 16 h at 37 °C. The MIC was determined to be the lowest concentration of the test compound, and it completely inhibited cell growth. Ampicillin and tetracycline were used as positive controls.

### 3.5. Gene Expression Analysis

For the analysis of *mraY* expression, overnight-cultured *S. aureus* 6538p was diluted with MHB medium and allowed to reach the mid-log phase through further incubation. Fourfold concentrations of the MIC of compounds **1**–**4** were introduced and incubated for 40 min at 37 °C. Lysostaphin was employed to enzymatically degrade the cell wall of *S. aureus*, followed by total RNA extraction using the easy-BLUE™ reagent (Intron Biotechnology, Seoul, Republic of Korea). Subsequently, cDNA was synthesized using the SuperScript III cDNA synthesis kit (Enzynomics, Seoul, Republic of Korea), and semi-quantitative RT–PCR was conducted with *mraY*-specific primers (*mraY* For and *mraY* Rev) (Table S1) under standard conditions. The DNA gyrase subunit A (*gyrA*) gene was utilized as a loading control.

### 3.6. MraY Molecular Cloning

The DNA sequence of the *S. aureus* 6538p *mraY* gene was amplified via PCR using specific primers (*mraY* For-NcoI and *mraY* Rev-XhoI for pET28a plasmid inserts; mraY For-SacI and mraY Rev-EcoRI for pRMC2 plasmid inserts) (Table S1), incorporating appropriate restriction enzyme sites for recombinant plasmid construction. The resulting recombinant plasmids containing *S. aureus* ATCC6538p *mraY*, namely, pET28a-*mraY* and pRMC2-*mraY*, were sequenced to confirm the inserts using the Sanger sequencing method. Plasmid pET28a-*mraY* was transformed into *E. coli* strain BL21(DE3), while plasmid pRMC2-*mraY* was transformed into *S. aureus* ATCC6538p using the *S. aureus* RN4220 strain as an intermediate to methylate DNA prior to electroporation [34].

### 3.7. MraY Enzyme Purification and Activity Assay

Recombinant MraY was prepared and purified by following previously described protocols [35]. Initially, *E. coli* strain BL21(DE3) containing the recombinant plasmid pET28a-*mraY* was cultured in LB media at 37 °C until reaching an absorbance of approximately 0.7 at 600 nm. Subsequently, 1 mM of IPTG (isopropyl β-D-1-thiogalactopyranoside) was added to induce protein expression and incubated for 16 h at 25 °C with agitation. Cells were harvested via centrifugation, and the membrane fraction was solubilized using a sodium phosphate–glycerol detergent buffer [35]. The solubilized crude extract was subjected to affinity chromatography using Ni^2+^-NTA-agarose resin, eluted with 250 mM imidazole to obtain purified MraY, and analyzed using SDS-PAGE.

Enzymatic activity assays for MraY were conducted according to previously described methods with slight modifications [36]. The UMP-Glo^TM^ glycosyltransferase assay (Promega Corporation, Madison, WI, USA) was performed according to the manufacturer’s instructions [25]. Then, 150 μM UDP-MurNAc-pentapeptide (Evitachem, Beijing, China) and 250 μM C55-P (Larodan AB, Solna, Sweden) were added to the Tris-HCl buffer system [36] and incubated with 300 μg/mL of purified MraY for 60 min at 37 °C. The luminescence resulting from the reaction was measured using a SpectraMax M3 multi-mode microplate reader (Molecular Devices, San Jose, CA, USA).

### 3.8. MraY Overexpression In Vivo

To induce the overexpression of tetracycline-inducible plasmids, a previously established method was employed [32]. *S. aureus* 6538p containing the recombinant plasmid pRMC2-*mraY* was cultured at 37 °C in MHB until reaching an absorbance of approximately 0.7 at 600 nm. Anhydrotetracycline was added to the culture at concentrations of 0.25, 0.5, and 1 μg/mL and incubated for 1 h. Following incubation, cells were harvested via centrifugation and diluted with MHB medium to achieve a concentration of approximately 5 × 10^6^ cfu/mL. Subsequently, the antibacterial activity of compounds **1**–**4** was evaluated as described above.

## 4. Conclusions

A marine-derived actinomycete (*Streptomyces* sp. MBTG32) exhibiting antibacterial activity against *Staphylococcus aureus* was investigated. The strain was identified using the 16S rDNA sequencing technique, and the isolated strain was closely related to *Streptomyces bacillaris*. Four tunicamycins, tunicamycin VII (**1**), tunicamycin VIII (**2**), corynetoxin U17a (**3**), and tunicamycin IX (**4**), were isolated from the ethyl acetate extract of the culture broth. The structures of the isolated compounds were elucidated based on comparisons of spectroscopic data with previously reported NMR data. Compounds **1**–**4** showed potent antibacterial activities against Gram-positive bacteria—especially *S. aureus*—with MIC values of 0.13–0.25 µg/mL. The inhibitory activities of these compounds against recombinant MraY, an essential enzyme that catalyzes the membrane-associated initial step of peptidoglycan synthesis, were assayed by using a discontinuous coupled assay system. The IC_50_ values of compounds **1**–**4** against MraY were 0.12, 0.13, 0.08, and 0.21 μg/mL, respectively. As MraY is an enzyme essential for the growth of *S. aureus*, the construction of deletion mutants of this gene could not be achieved. To overcome this limitation, the effects of *mraY* overexpression in *S. aureus* cells on the MICs of compounds **1**–**4** were evaluated. Overexpression studies showed that the MICs of compounds **1**–**4** increased up to 32-fold, indicating that the antibacterial activity of these compounds is associated with the inhibition of MraY. Therefore, we confirmed that the marine-derived tunicamycins reported here are strong MraY inhibitors, effectively inhibiting the growth of the selected MRSA strains. As MraY inhibitors are a promising novel target for drug-resistant Gram-positive bacteria, the overexpression system used in this study could be an additional tool for evaluating in vivo activity of MraY inhibitors.

## Figures and Tables

**Table 1:** Results of the antimicrobial activities of compounds **1**–**4**.

Compound	MIC (μg/mL)					
	Gram (+) Bacteria			Gram (−) Bacteria		
	A	B	C	D	E	F
**1**	0.13	2	2	64	>128	>128
**2**	0.13	2	2	>128	>128	>128
**3**	0.06	1	2	64	>128	>128
**4**	0.25	4	8	>128	>128	>128
Ampicillin	0.13	1	0.5	0.25	128	8
Tetracycline	0.13	0.25	0.25	0.25	0.25	0.5

A: *Staphylococcus aureus* ATCC6538p, B: *Enterococcus faecalis* ATCC19433, C: *Enterococcus faecium* ATCC19434, D: *Salmonella enterica* ATCC14028, E: *Klebsiella pneumoniae* ATCC10031, F: *Escherichia coli* ATCC25922.

**Table 2:** Antibacterial activities of compounds **1**–**4** against MSSA and MRSA strains.

Strain	MIC (μg/mL)					
	Dap	Van	1	2	3	4
CCARM0027 ^a^	8	0.25	32	64	8	32
CCARM0204 ^a^	2	0.13	0.06	0.13	0.06	0.13
CCARM0205 ^a^	2	0.13	0.13	0.13	0.06	0.25
CCARM3640 ^a^	8	0.25	16	32	8	16
CCARM3089 ^b^	>32	1	>128	>128	>128	>128
CCARM3090 ^b^	>32	0.5	8	8	2	8
CCARM3634 ^b^	>32	0.5	32	32	16	32
CCARM3635 ^b^	>32	1	>128	64	32	>128
ATCC43300 ^b^	>32	1	32	>128	16	>128
ATCC700787 ^b^	>32	1	16	32	8	16
ATCC700788 ^b^	>32	1	>128	>128	>128	>128

^a^ Methicillin-sensitive *Staphylococcus aureus* (MSSA). ^b^ Methicillin-resistant *Staphylococcus aureus* (MRSA). Dap: daptomycin, Van: vancomycin.

## Data Availability

All data are contained within this article and the Supplementary Materials.

## Supplementary Materials

The following supporting information can be downloaded at https://www.mdpi.com/article/10.3390/md22070293/s1, Figure S1: ^1^H NMR (600 MHz, MeOH-*d_4_*) spectrum of **1**; Figure S2: ^13^C NMR (150 MHz, MeOH-*d_4_*) spectrum of **1**; Figure S3: ^13^C NMR (150 MHz, DMSO-*d_6_*) spectrum of **1**; Figure S4: ^1^H NMR (600 MHz, DMSO-*d_6_*) spectrum of **2**; Figure S5: ^13^C NMR (600 MHz, DMSO-*d_6_*) spectrum of **2**; Figure S6: ^1^H NMR (600 MHz, DMSO-*d_6_*) spectrum of **3**; Figure S7: ^13^C NMR (600 MHz, DMSO-*d_6_*) spectrum of **3**; Figure S8: ^1^H NMR (600 MHz, DMSO-*d_6_*) spectrum of **4**; Figure S9: ^13^C NMR (600 MHz, DMSO-*d_6_*) spectrum of **4**; Figure S10: HR-ESI-MS data of **1**; Figure S11: HR-ESI-MS data of **2**; Figure S12: HR-ESI-MS data of **3**; Figure S13: HR-ESI-MS data of **4**; Figure S14: HR-ESI-MS fragmentation analysis of **1**; Figure S15: HR-ESI-MS fragmentation analysis of **2**; Figure S16: HR-ESI-MS fragmentation analysis of **3**; Figure S17: HR-ESI-MS fragmentation analysis of **4**; Table S1: List of oligonucleotides used in this study; Table S2: ^13^C NMR comparison of **1** with tunicamycin V in MeOH-*d_4_*; Table S3: ^13^C NMR comparison of **3** with corynetoxin U17a in DMSO-*d_6_*.
