# Supplementary material for: Tunicamycins from Marine-Derived Streptomyces bacillaris Inhibit MurNAc-Pentapeptide Translocase in Staphylococcus aureus
Source: Mar Drugs. 2024 Jun 26;22(7):293. doi: 10.3390/md22070293 (PMC11277991; doi:10.3390/md22070293)
Supplement: Supplementary file 1 [file marinedrugs-22-00293-s001.zip › marinedrugs-3028275-supplementary.pdf]

Supporting Information

Tunicamycins from Marine-Derived *Streptomyces bacillaris* Inhibit MurNAc-Pentapeptide Translocase in *Staphylococcus aureus*

Jayho Lee [1], Ji-Yeon Hwang [2], Daehyun Oh [3], Dong-Chan Oh [2], Hyeung-geun Park [3], Jongheon Shin [2,*] and Ki-Bong Oh [1,*]

[1] Department of Agricultural Biotechnology, College of Agriculture and Life Sciences and Natural Products Research Institute, Seoul National University, Seoul 08826, Republic of Korea; jayho@snu.ac.kr

[2] Natural Products Research Institute, College of Pharmacy, Seoul National University, Seoul 08826, Republic of Korea; yahyah7@snu.ac.kr (J.-Y.H.); dongchanoh@snu.ac.kr (D.-C.O.)

[3] Research Institute of Pharmaceutical Sciences, College of Pharmacy, Seoul National University, Seoul 08826, Republic of Korea; oh1see@snu.ac.kr (D.O.); hgpk@snu.ac.kr (H.-g.P.)

* Correspondence: shinj@snu.ac.kr (J.S.); ohkibong@snu.ac.kr (K.-B.O.); Tel.: +82-2-880-2484 (J.S.); +82-2-880-4646 (K.-B.O.)

Contents

Figure S1. ^{1}H NMR (600 MHz, MeOH-d_4) spectrum of **1**

Figure S2. ^{13}C NMR (150 MHz, MeOH-d_4) spectrum of **1**

Figure S3. ^{13}C NMR (150 MHz, DMSO-d_6) spectrum of **1**

Figure S4. ^{1}H NMR (600 MHz, DMSO-d_6) spectrum of **2**

Figure S5. ^{13}C NMR (600 MHz, DMSO-d_6) spectrum of **2**

Figure S6. ^{1}H NMR (600 MHz, DMSO-d_6) spectrum of **3**

Figure S7. ^{13}C NMR (600 MHz, DMSO-d_6) spectrum of **3**

Figure S8. ^{1}H NMR (600 MHz, DMSO-d_6) spectrum of **4**

Figure S9. ^{13}C NMR (600 MHz, DMSO-d_6) spectrum of **4**

Figure S10. HR-ESI-MS data of **1**

Figure S11. HR-ESI-MS data of **2**

Figure S12. HR-ESI-MS data of **3**

Figure S13. HR-ESI-MS data of **4**

Figure S14. HR-ESI-MS fragmentation analysis of **1**

Figure S15. HR-ESI-MS fragmentation analysis of **2**

Figure S16. HR-ESI-MS fragmentation analysis of **3**

Figure S17. HR-ESI-MS fragmentation analysis of **4**

Table S1. List of oligonucleotides used in this study

Table S2. ^{13}C NMR comparison of **1** with tunicamycin V in MeOH-d_4

Table S3. ^{13}C NMR comparison of **3** with corynetoxin U17a in DMSO-d_6

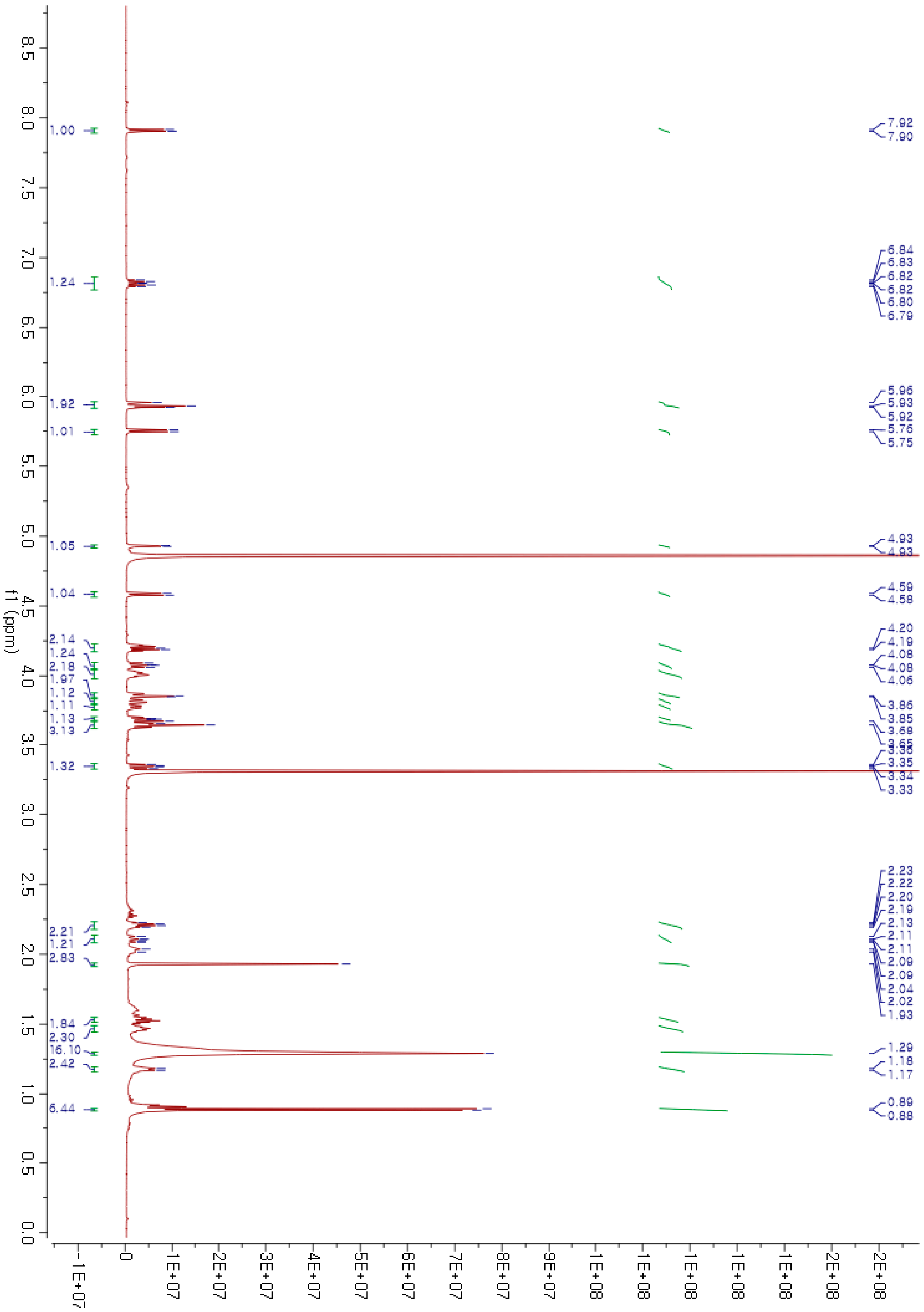

Figure S1. ^{1}H NMR (600 MHz, MeOH-d_4) spectrum of **1**

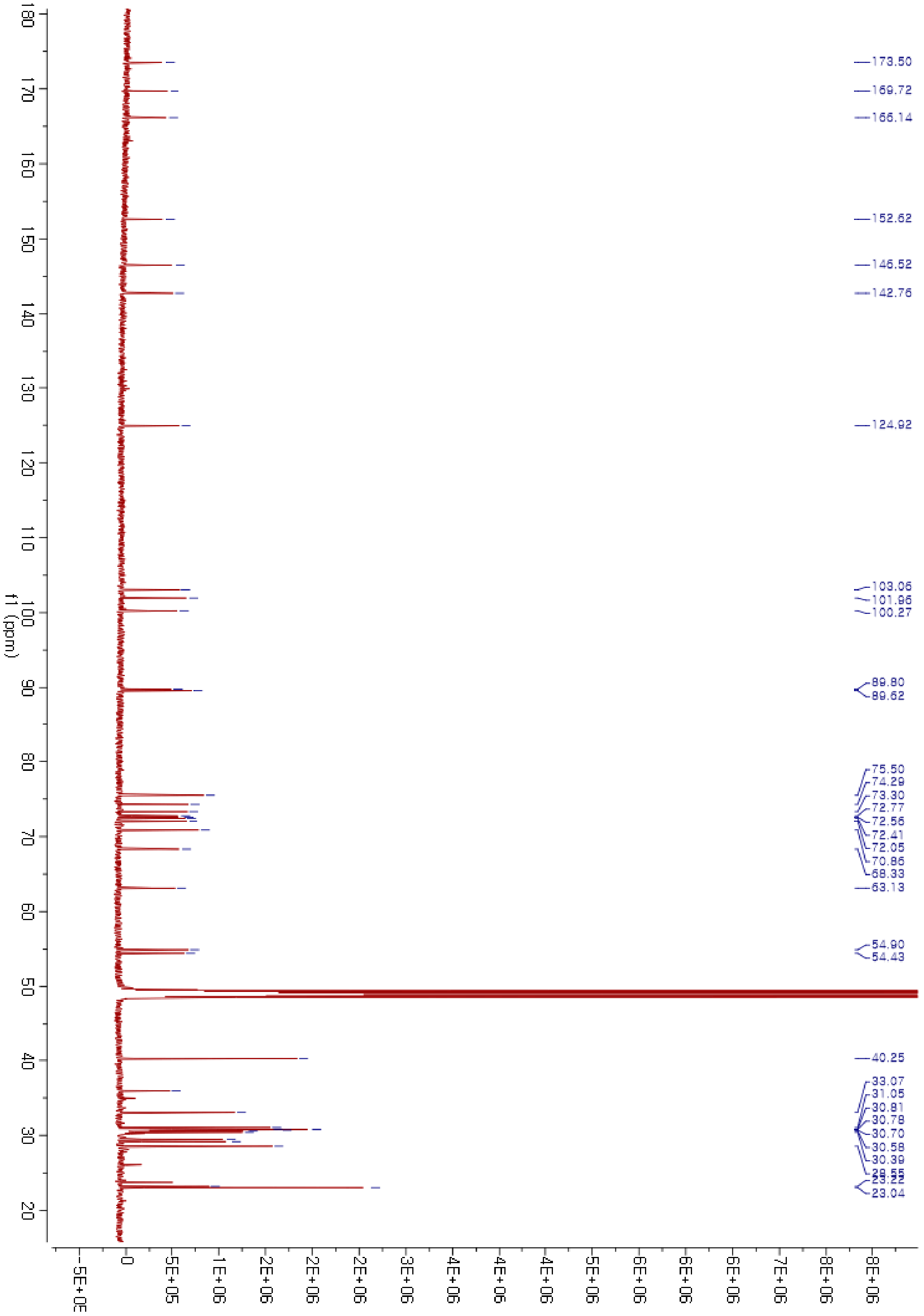

Figure S2. ^{13}C NMR (150 MHz, MeOH-d_4) spectrum of **1**

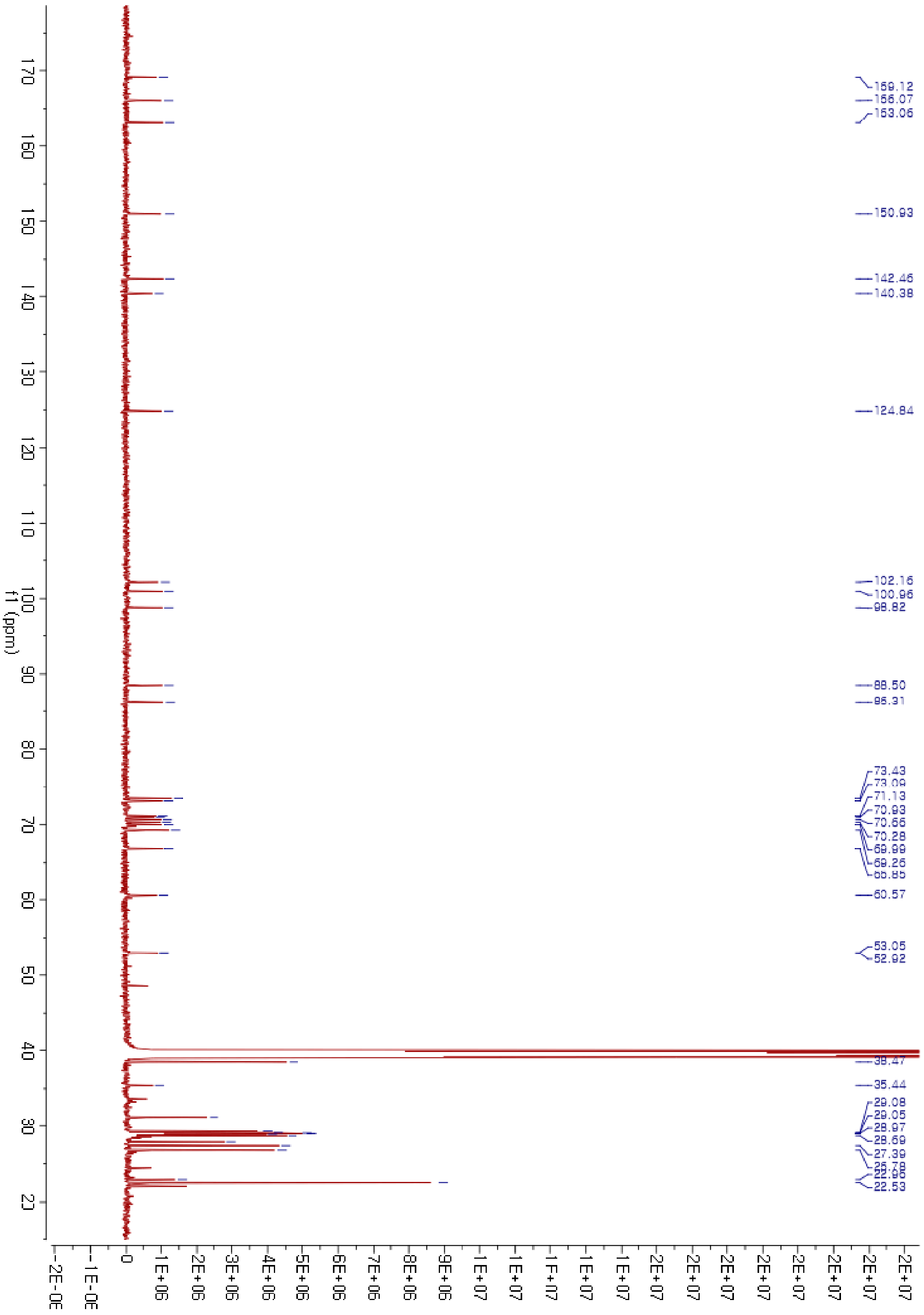

Figure S3. ^{13}C NMR (150 MHz, DMSO-d_6) spectrum of **1**

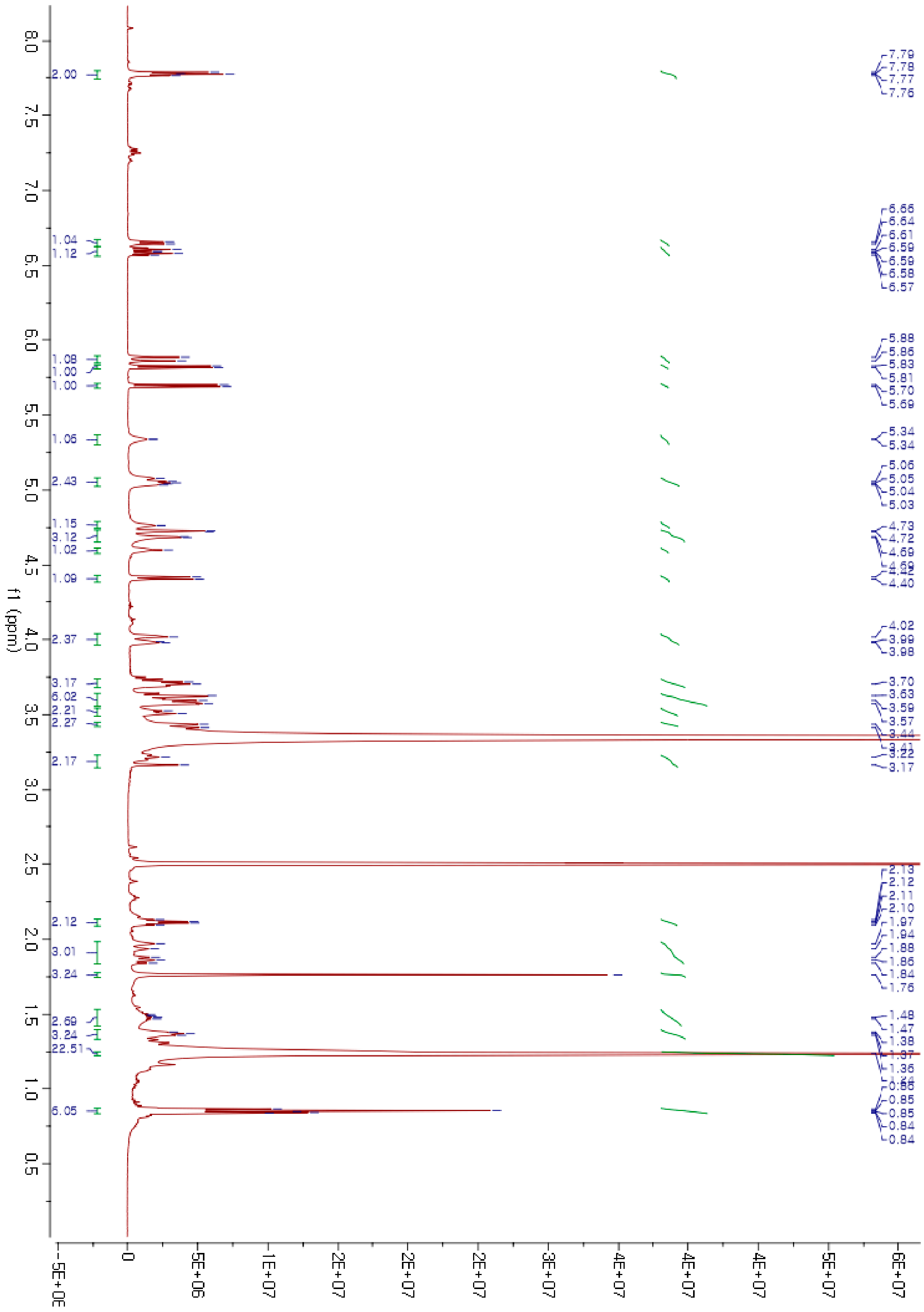

Figure S4. ^{1}H NMR (600 MHz, DMSO-d_6) spectrum of **2**

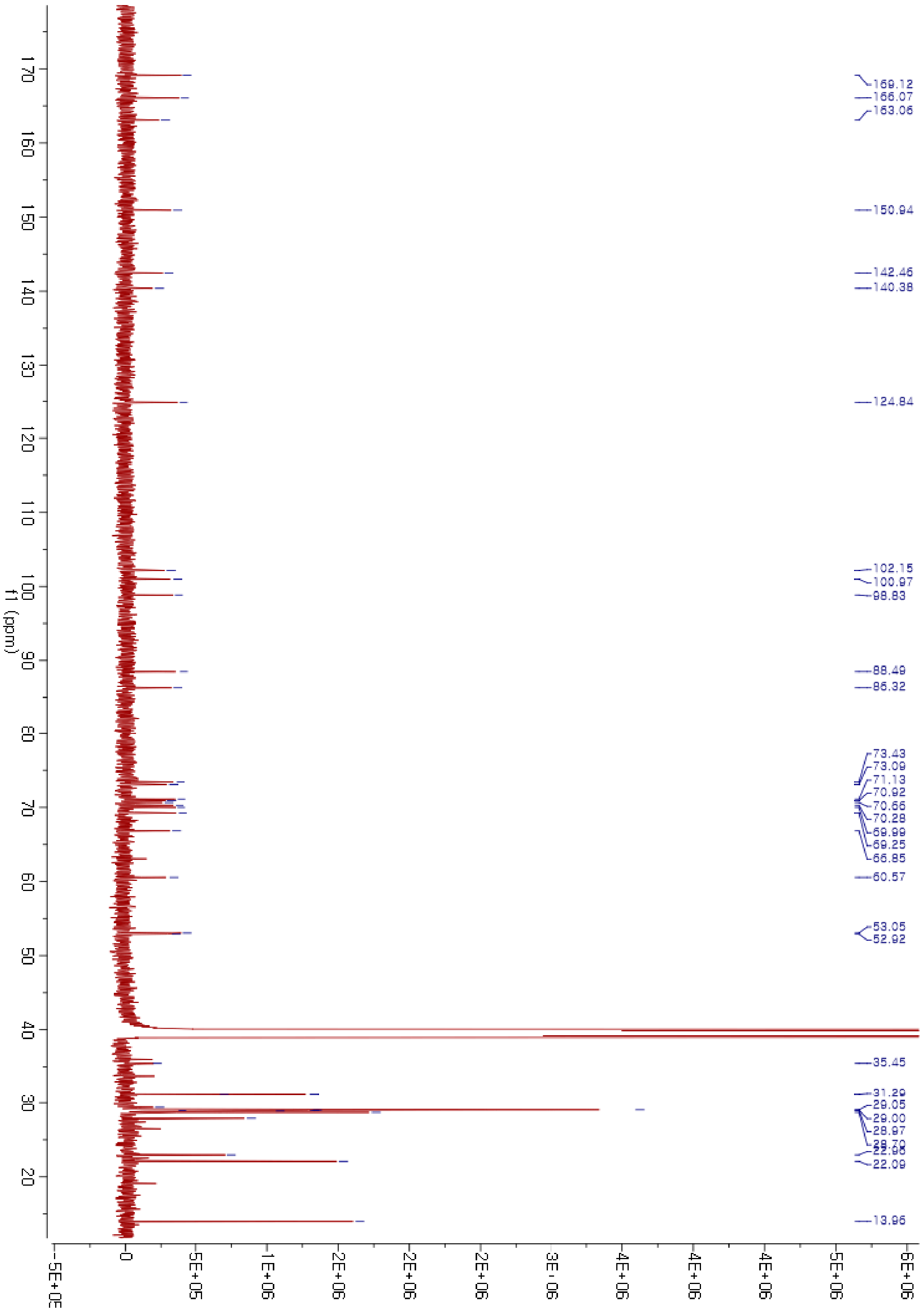

Figure S5. ^{13}C NMR (150 MHz, DMSO-d_6) spectrum of **2**

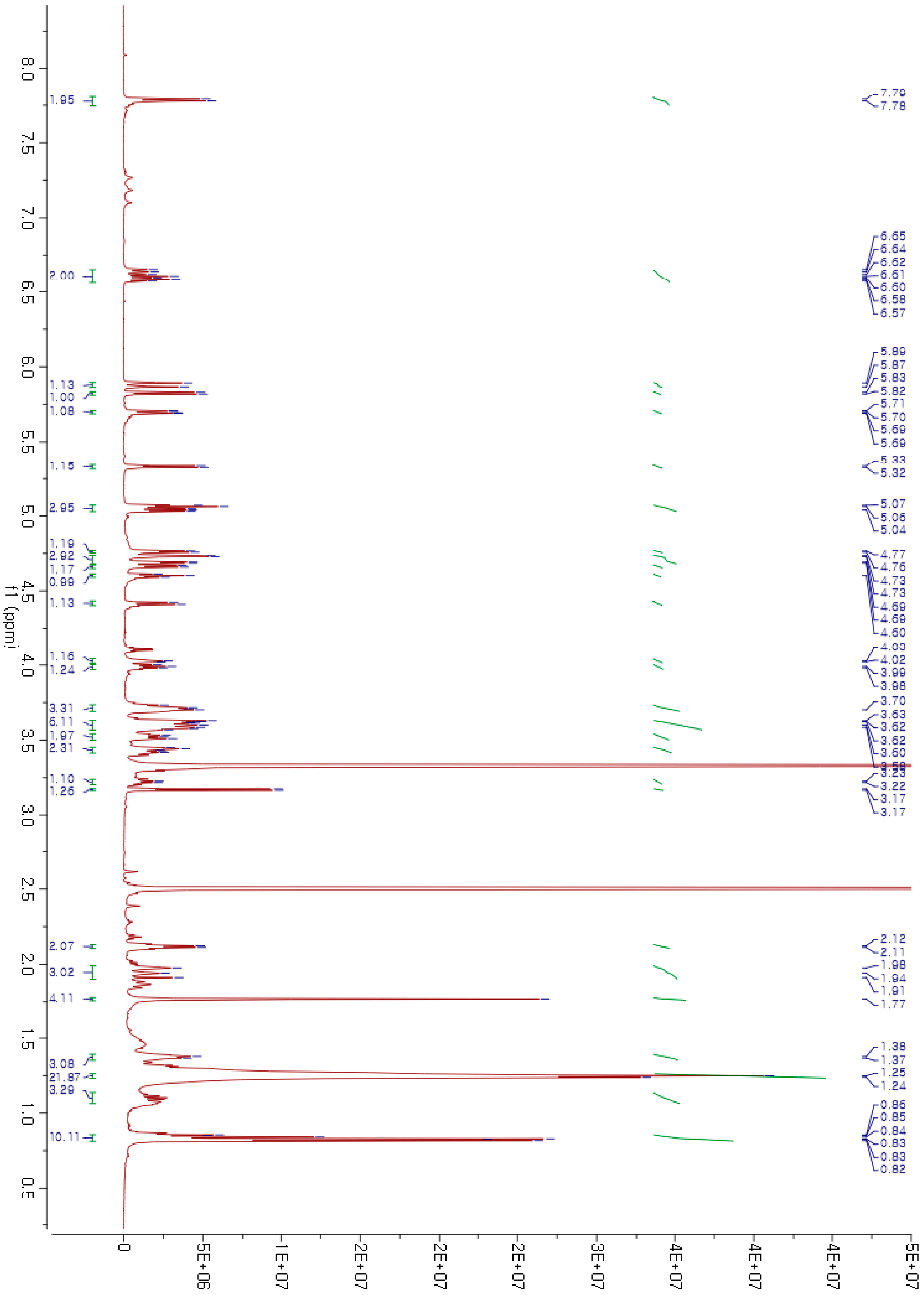

Figure S6. ^{1}H NMR (600 MHz, DMSO-d_6) spectrum of **3**

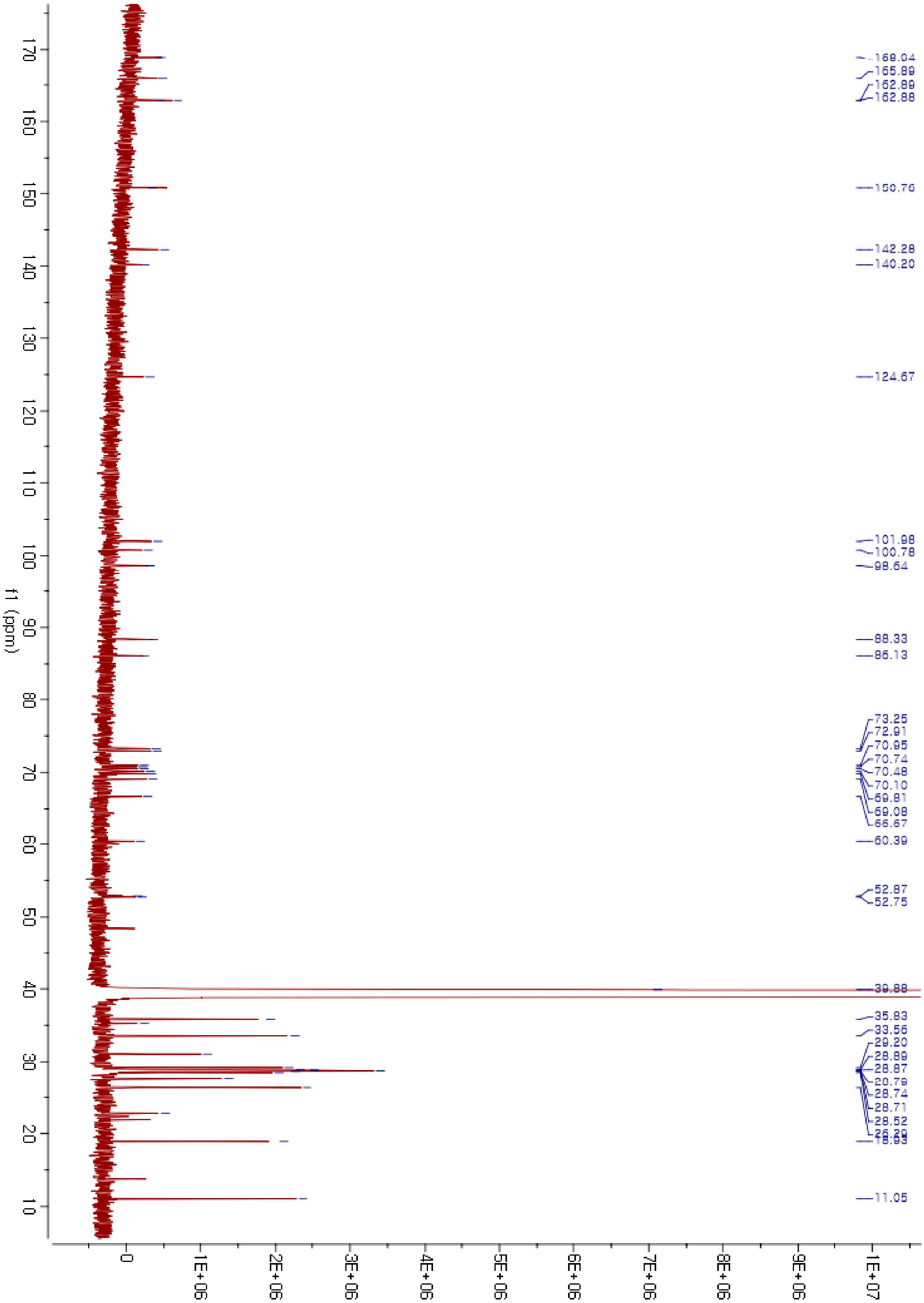

Figure S7. ^{13}C NMR (150 MHz, DMSO-d_6) spectrum of **3**

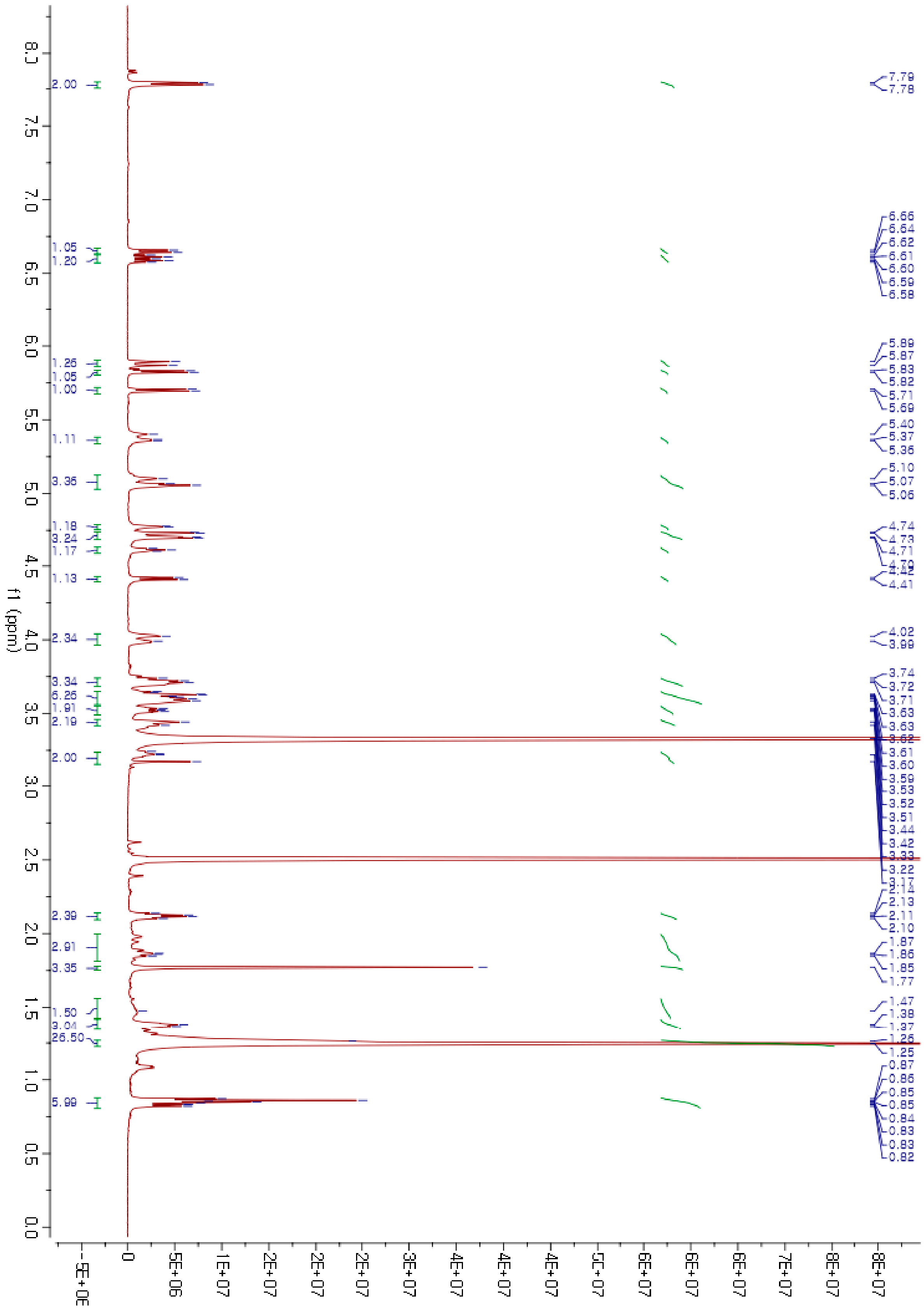

Figure S8. ^{1}H NMR (600 MHz, DMSO-d_6) spectrum of **4**

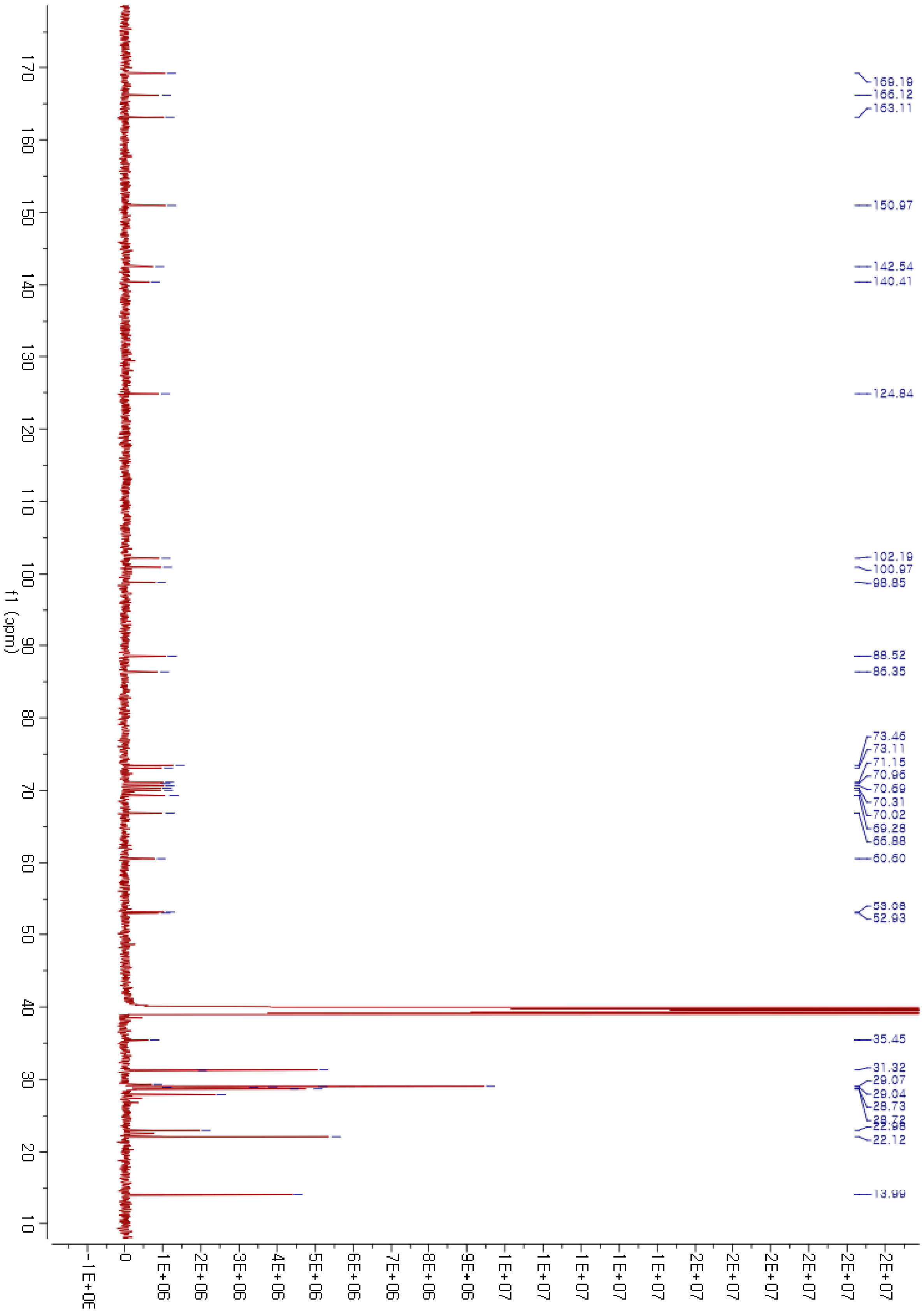

Figure S9. ^{13}C NMR (150 MHz, DMSO-d_6) spectrum of **4**

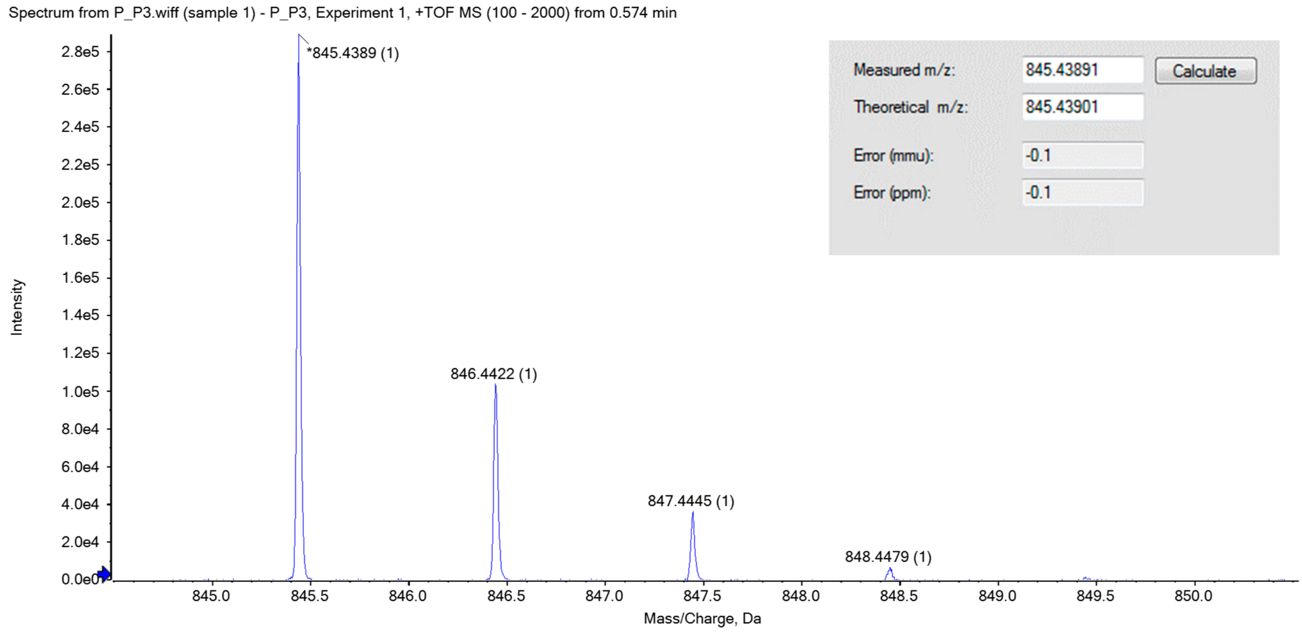

Figure S10. HR-ESI-MS data of **1**

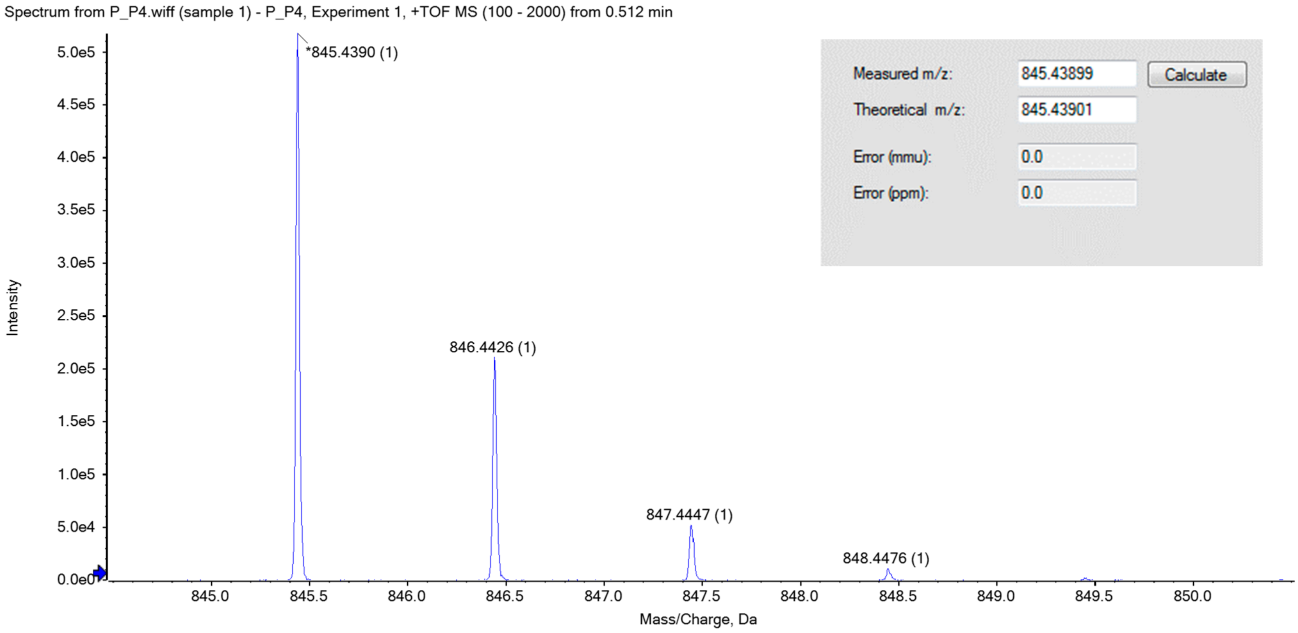

Figure S11. HR-ESI-MS data of **2**

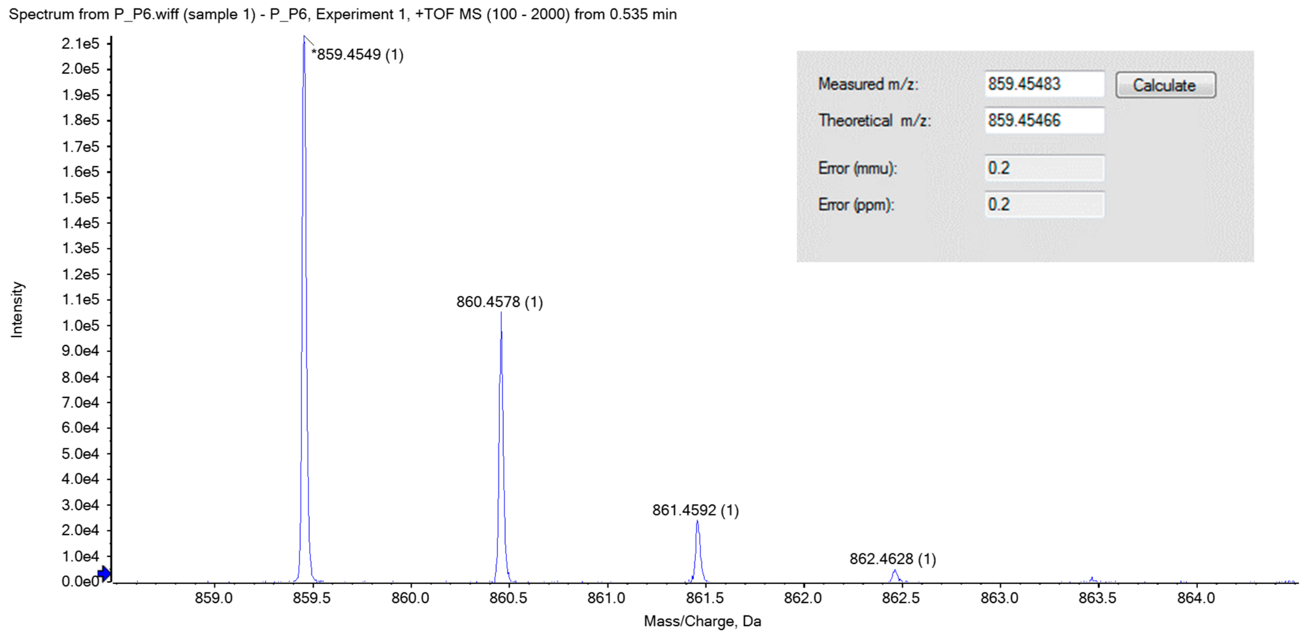

Figure S12. HR-ESI-MS data of **3**

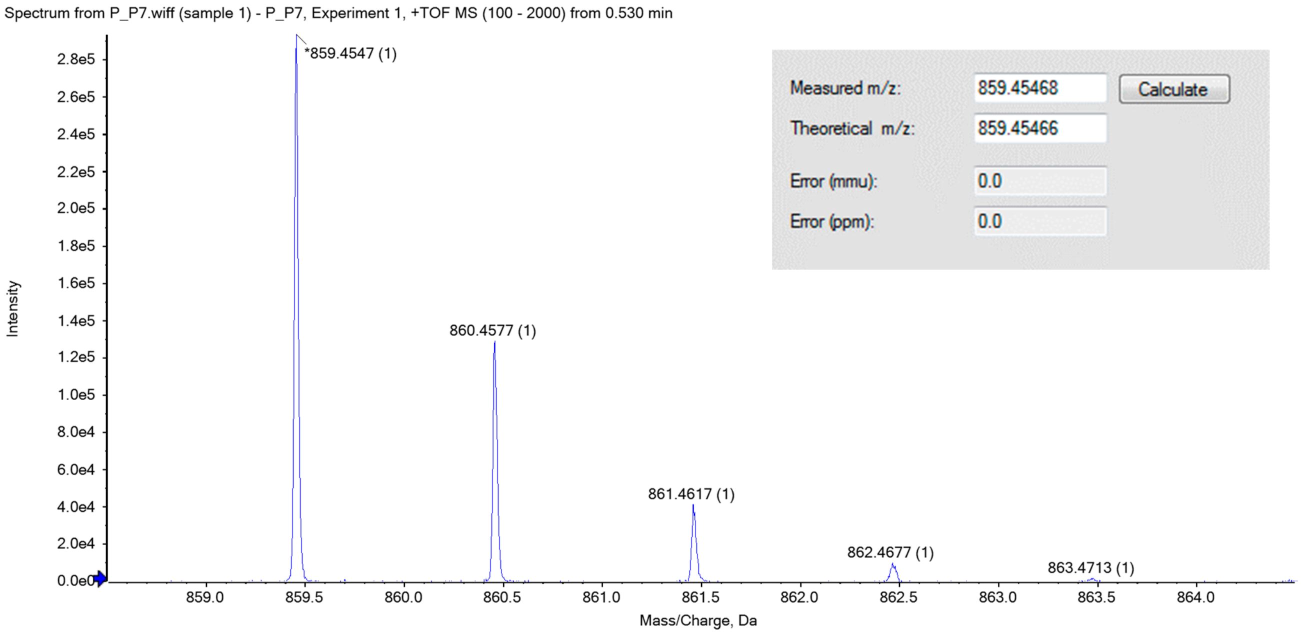

Figure S13. HR-ESI-MS data of **4**

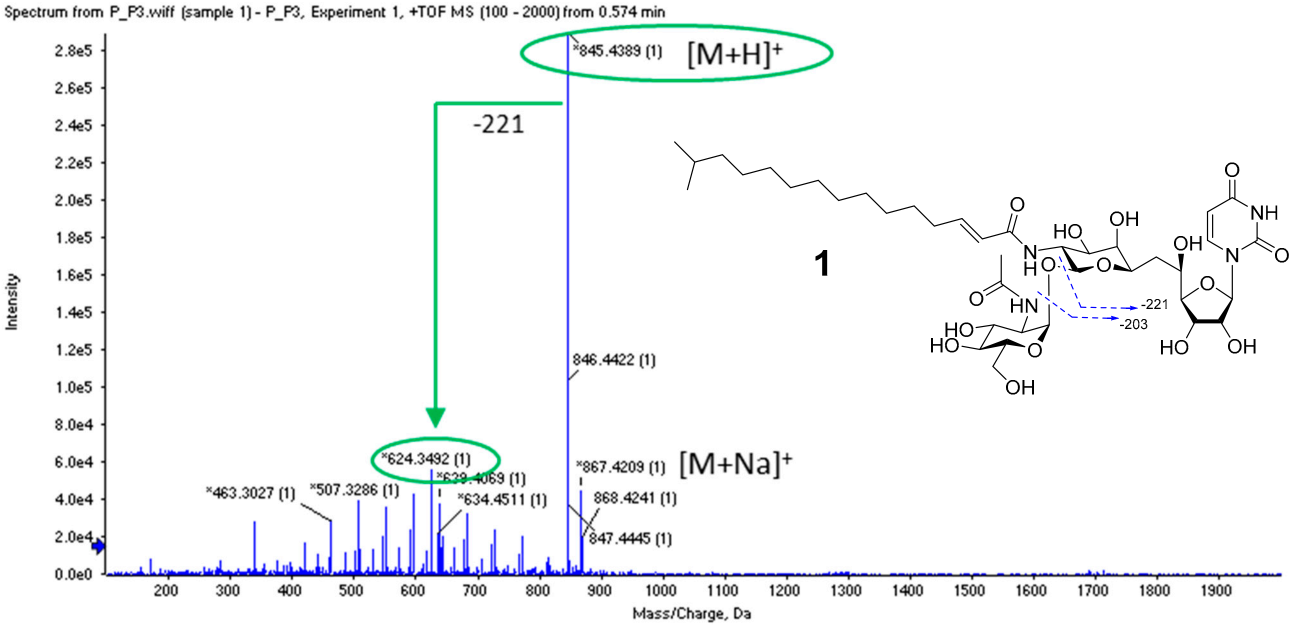

Figure S14. HR-ESI-MS fragmentation analysis of **1**

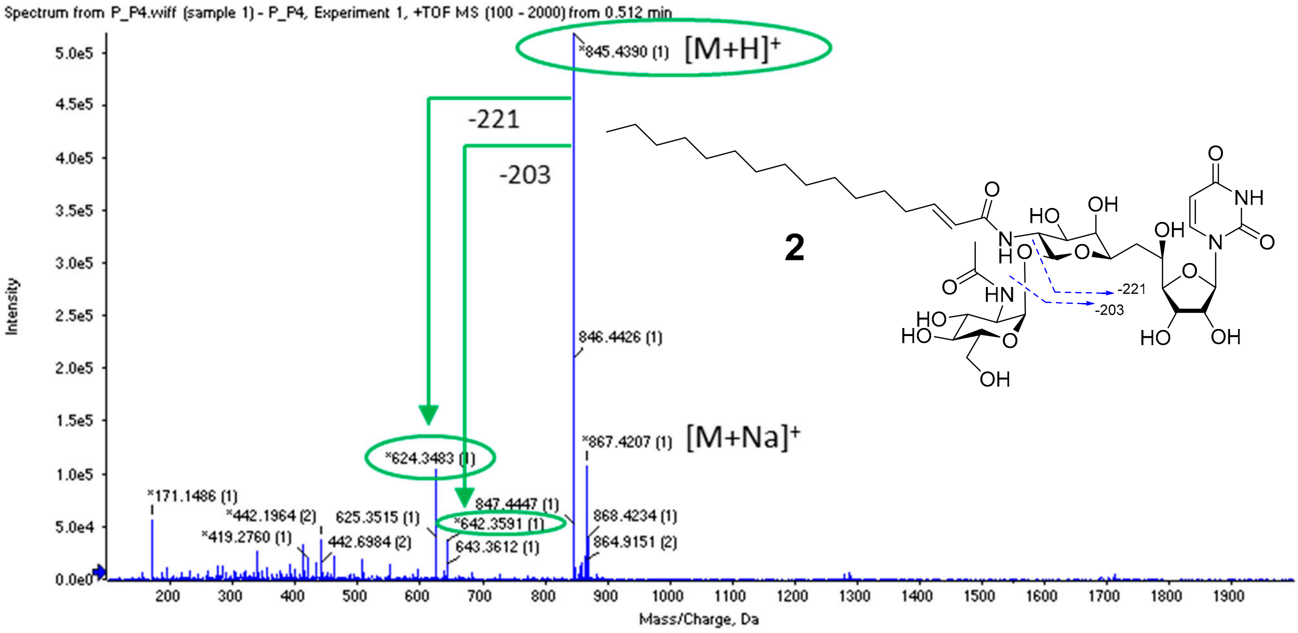

Figure S15. HR-ESI-MS fragmentation analysis of **2**

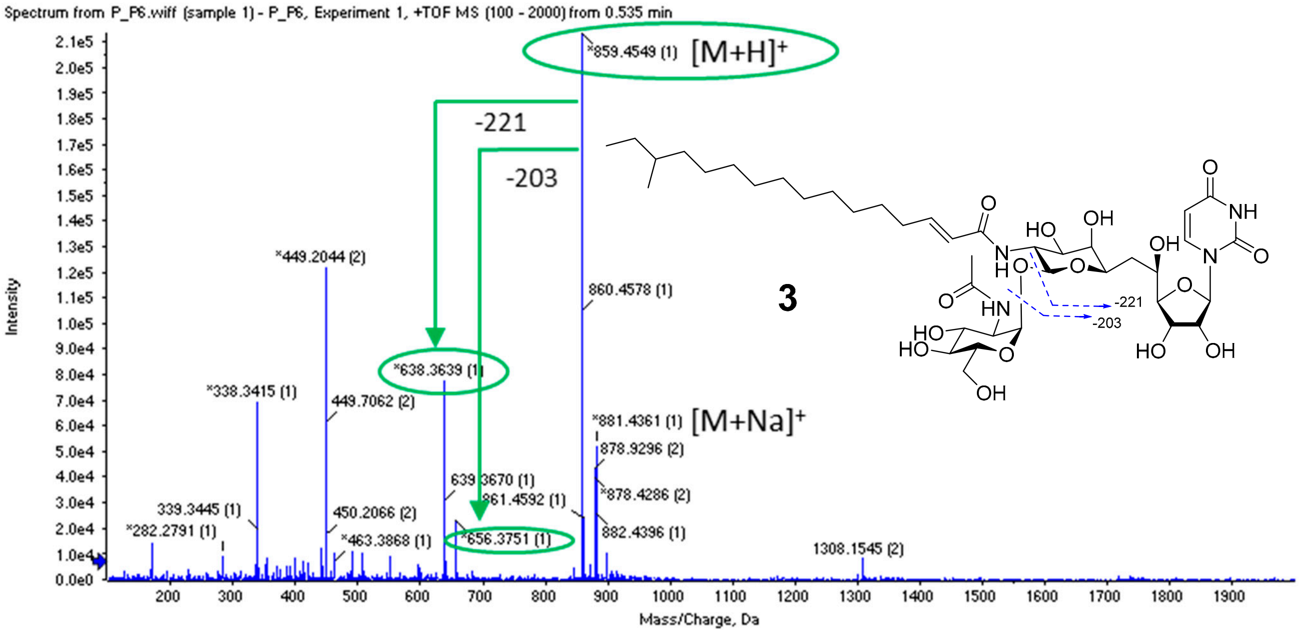

Figure S16. HR-ESI-MS fragmentation analysis of **3**

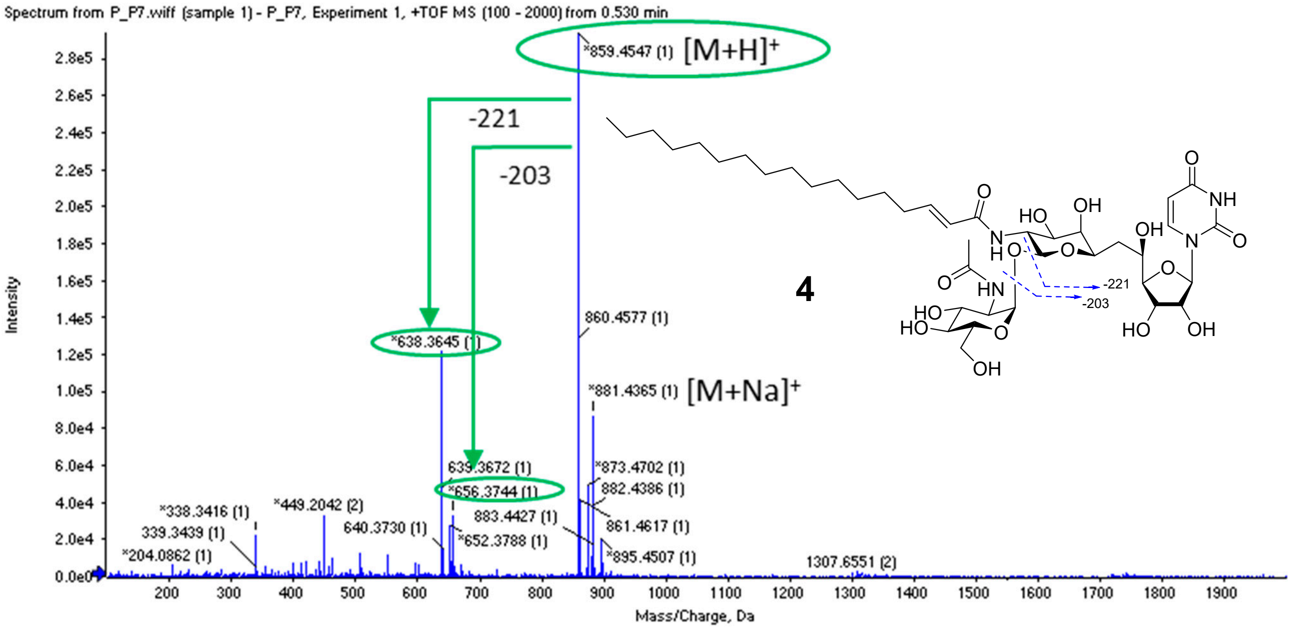

Figure S17. HR-ESI-MS fragmentation analysis of **4**

Table S1. List of oligonucleotides used in this study

Primer Name	Sequence
27F	5′-AGAGTTTGATCCTGGCTCAG-3′
1429R	5′-GGTTACCTTGTTACGACTT-3′
mraY For	5′-TGGTGAATTTTTCTACGAGC-3′
mraY Rev	5′-CCATAAACACTTTAGCAGGG-3′
gyrA For	5′-CGTGACAAGAAAATTGACGG-3′
gyrA Rev	5′- TGACACTAGCATTTGCATCC -3′
mraY For-NcoI	5′-GA**CCATGG**AGATGATTTTTGTATATGCGTT-3′
mraY Rev-XhoI	5′-TT**CTCGAG**ATGCACTCCAATCCATAAAC-3′
mraY For-SacI	5′-TAA**GAGCTC**ATGATTTTTGTATATGCGTTAT-3
mraY Rev-EcoRI	5′-GT**GAATTC**TTAATGCACTCCAATCCATAAAC-3′

Restriction enzyme sites are indicated as bold.

Table S2. ^{13}C NMR comparison of **1** with tunicamycin V in MeOH-d_4.

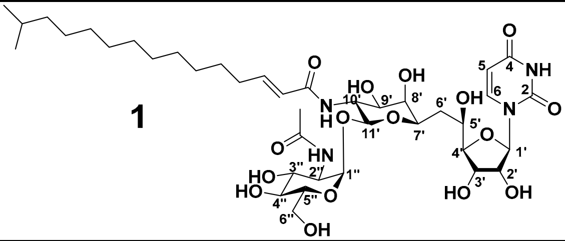

1

structural motif	carbon residue	tunicamycin V [1]	compound 1	Dev.
Uracil	C-2	152.6	152.6	0
	C-4	166.1	166.1	0
	C-5	103.1	103.1	0
	C-6	142.8	142.8	0
tunicamine	C-1′	89.9	89.8	0.1
	C-2′	75.5	75.5	0
	C-3′	70.9	70.9	0
	C-4′	89.6	89.6	0
	C-5′	68.4	68.3	0.1
	C-6′	35.93	35.93	0
	C-7′	72.6	72.6	0
	C-8′	72.1	72.1	0
	C-9′	73.3	73.3	0
	C-10′	54.5	54.4	0.1
	C-11′	102.0	102.0	0
GlcNAc	C-1′′	100.3	100.3	0
	C-2′′	54.9	54.9	0
	C-3′′	72.8	72.8	0
	C-4′′	72.5	72.4	0.1
	C-5′′	74.3	74.3	0
	C-6′′	63.2	63.1	0.1
	C=O	173.5	173.5	0
	CH_3	23.2	23.2	0
N-acyl chain	C=O	169.7	169.7	0
	C-2′′′	124.9	124.9	0
	C-3′′′	146.5	146.5	0
	C-4′′′	33.1	33.1	0
	C-5′′′	29.5	29.5	0
	$(CH_2)_n$	31.1	31.1	0
		30.8	30.8	0
			30.8	
		30.7	30.7	0
		30.6	30.6	0
		30.4	30.4	0
		28.6	28.6	0
	ω-CH_2	40.3	40.3	0
	CH	29.2	29.2	0
	CH_3′s	23.0	23.0	0
		23.0	23.0	0

Table S3. ^{13}C NMR comparison of **3** with corynetoxin U17a in DMSO-d_6

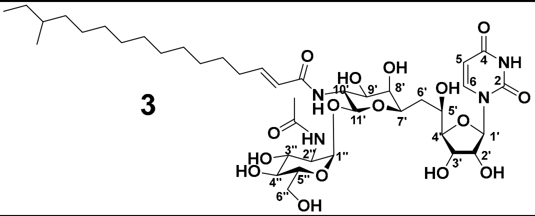

structural motif	carbon residue	corynetoxin U17a [2]	compound 3	Dev.
Uracil	C-2	150.8	150.8	0.0
	C-4	162.9	162.9	0.0
	C-5	102.0	102.0	0.0
	C-6	140.2	140.2	0.0
tunicamine	C-1′	86.4	86.1	0.3
	C-2′	73.3	73.3	0.0
	C-3′	70.2	70.1	0.1
	C-4′	88.3	88.3	-0.1
	C-5′	66.8	66.7	0.1
	C-6′	35.2	35.3	0.0
	C-7′	71.1	71.0	0.1
	C-8′	70.0	69.8	0.2
	C-9′	69.1	69.1	0.0
	C-10′	53.0	52.9	0.1
	C-11′	98.6	98.6	0.0
GlcNAc	C-1″	100.8	100.8	0.0
	C-2″	52.9	52.8	0.1
	C-3″	70.6	70.5	0.1
	C-4″	70.8	70.7	0.1
	C-5″	72.9	72.9	0.0
	C-6″	60.6	60.4	0.2
	C=O	169.0	168.9	0.0
	CH_3	22.8	22.8	0.0
N-acyl chain	C=O	166.0	165.9	0.1
	C-2‴	124.7	124.7	0.1
	C-3‴	142.3	142.3	0.0
	C-4‴	31.0	31.1	0.0
	C-5‴	26.3	26.3	0.0
	C-6‴	28.7	28.7	0.0
	C-7‴	28.9	28.9	0.0
	C-8‴	28.9	28.9	0.0
	C-9‴	28.8	28.8	0.0
	C-10‴	28.8	28.7	0.0
	C-11‴	28.5	28.5	0.0
	C-12‴	27.7	27.7	0.0
	C-13‴	35.8	35.8	0.0
	C-14‴[F]	39.6	39.9	-0.3
	C-15‴[F]	29.2	29.2	0.0
	C-16‴[F]	11.0	11.1	-0.1
	C-17‴[F]	18.9	18.9	0.0

[1] Li, J.; Yu, B. A modular approach to the total synthesis of tunicamycins. *Angew. Chem. Int. Ed. Engl.* **2015**, *54*, 6618-6621, doi:10.1002/anie.201501890.
[2] Frahn, J.L.; Edgar, J.A.; Jones, A.J.; Cockrum, P.A.; Anderton, N.A.; Culvenor, C.C.J. Structure of the corynetoxins, metabolites of *Corynebacterium rathayi* responsible for toxicity of annual ryegrass (*Lolium rigidum*) pastures. *Aust. J. Chem.* **1984**, *37*, 165-182, doi:10.1071/Ch9840165.
